# Cuticular property affects the insecticidal synergy of major constituents in thyme oil against houseflies, *Musca domestica*

**DOI:** 10.1038/s41598-023-39898-6

**Published:** 2023-08-04

**Authors:** Junho Yoon, Jun-Hyung Tak

**Affiliations:** 1 Department of Agricultural Biotechnology, Seoul National University, Seoul, 08826 South Korea; 2 Research Institute of Agriculture and Life Sciences, Seoul National University, Seoul, 08826 South Korea

**Keywords:** Mechanism of action, Natural products, Pharmacology

## Abstract

Plant essential oils are intricate blends comprising predominantly of monoterpenes and some sesquiterpenes. These oils display diverse bioactivities against targeted organisms, often arising from complex interactions among their constituents, which may demonstrate synergistic or antagonistic effects. Despite their wide use as botanical insecticides, the mechanisms behind these interactions and their effects on bioactivity are poorly understood. This study investigated the synergistic interaction of thymol and *p*-cymene, two major constituents of *Thymus vulgaris* essential oil, on the larvae and adults of the housefly, *Musca domestica*. The results showed that *p*-cymene synergized the insecticidal activity of thymol in adult houseflies, but not in larvae. GC–MS analyses and bioassays indicated the increased cuticular penetration of thymol by *p*-cymene was the mechanism of synergy, which was observed only in the adults. Two potential routes were proposed: the expansion of the wetting area, or the disruption of cuticular integrity through dissolving the wax layer. The sequential application and large-volume treatment bioassay results suggested that the former was the more likely mechanism. Also, the hydrophobicity of the cuticle seemed critical for this stage-specific synergy. Wax-devoid adults failed to show synergistic toxicity, whereas artificially wax-coated larvae gained a synergistic effect. Overall, the findings provide insights into the synergistic mechanism of insecticidal activity of plant essential oils and suggest potential applications in developing effective strategies using penetration-enhancing synergists.

## Introduction

Increasing concerns about the environmental and health impacts of synthetic insecticides have inspired the search for safer pest control strategies, and plant essential oils are considered as alrternative candidates. They seem promising for controlling various insect pests, as they are generally considered less toxic to humans and wild animals than conventional pesticides^1^. Nowadays, many commercialized botanical insecticides are available on the market^2–5^. Most essential oils are extracted from the flowering tops, bark, resin, and seeds from various plant sources via steam distillation^6^. They are composed of various constituents, mostly monoterpenes and sesquiterpenes^7^. The chemical complexity and structural diversity of essential oil constituents often result in a combined bioactivity that is greater or lesser than those of the individual components; however, the mechanism of these intermolecular interactions is less understood.

Many oils induce a rapid onset of poisoning responses in insects, which suggests that the active constituents of these oils may directly influence the nervous system and exhibit insecticidal activity^8–10^. Studies have shown that the structural diversity of the major compounds in essential oils may indicate multiple modes of action. For example, thymol and nootkatone, the major constituents of thyme and cedarwood oils, respectively, modulate the gamma-aminobutyric acid (GABA) receptors of *Drosophila melanogaster* in opposing ways^11,12^. Other neural targets, including octopaminergic, tyraminergic, and cholinergic systems, have also been proposed as potential target sites/systems for various essential oil-derived compounds^13–16^.

Several hypotheses have been proposed regarding the synergistic mechanism of plant essential oils. Similar to conventional synergists, basil and geranium oils inhibit the activity of both cytochrome P450s and glutathione-S-transferase, which are the major detoxification enzymes for toxicants^17^. In another study, limonene amplified the electrophysiological response to estragole in the central nervous system of *Spodoptera litura* larvae^18^. Against other lepidopteran insects such as *Trichoplusia ni*, changing the cuticular penetration of active compounds by lowering the surface tension of the mixture has been proposed as another potential synergistic mechanism^3,19^.

The housefly, *Musca domestica* L. is a cosmopolitan insect pest vector of serious diseases, including typhoid, dysentery, diphtheria, leprosy, tuberculosis, and intestinal parasites^20^. Like many other insects, the housefly undergoes complete metamorphosis, where its habitat, physiology, and behavior are distinctively stage specific. The larvae occupy damp habitats, such as carcasses, manure, or garbage. This may be related to a lack of cuticular hydrophobicity^21,22^. In fact, the larvae actively favor humid conditions to avoid dehydration^23^. Adult houseflies are also susceptible to water loss and develop a hydrophobic wax layer composed of long-chain hydrocarbons (> C_20_) to prevent water loss^24,25^.

In the present study, stage-specific insecticidal synergy was examined in both adult and larval houseflies. We hypothesized that increased cuticular penetration would affect the toxicity of the binary mixture of thymol and *p*-cymene, and that the difference in cuticular composition would affect permeation and toxicity. We conducted comparative studies on larvae and adults of insecticide-susceptible houseflies and provided evidence that the combination of treated compounds and surface properties of the target pest is critical for exerting synergy.

## Results

### Chemical composition of thyme oil

A total of 83.6% of the thyme oil constituents were identified by GC–MS analysis, and *p*-cymene was the most abundant compound (38.0%), followed by thymol (31.8%) (Supplementary Table S1). Linalool (4.1%) and α-pinene (3.1%) were also present in the oil but constituted notably less than the two primary compounds. These four major compounds were evaluated for their roles in the overall toxicity of the oil against houseflies.

### Insecticidal activity of thyme oil and its major constituents via topical application

By topical application, the insecticidal activity of thyme oil was represented by an LD_50_ of 83.1 μg/insect for adults and 86.0 μg/insect for larvae, while the positive control permethrin had an LD_50_ of 0.23 and 25.76 μg/insect for adults and larvae, respectively (Table 1). No mortality was observed in the acetone-treated negative control.

**Table 1:** Insecticidal activity of thyme oil and permethrin against adult and larval houseflies via topical application.

Stage	Material	LD_50_ (μg/insect, 95% CI^†^)	LD_90_ (μg/insect, 95% CI^†^)	Slope (± SE)	*χ*^*2*^	*d.f.*^‡^	*p*
Adult	Thyme oil	83.1 (70.0–98.8)	267.2 (205.4–392.2)	2.5 (± 0.3)	13.3	22	0.92
	Permethrin	0.2 (0.1–0.4)	3.6 (1.8–10.8)	1.0 (± 0.1)	12.6	16	0.70
Larva	Thyme oil	86.0 (69.0–108.4)	386.3 (271.5–648.1)	5.4 (± 0.6)	4.4	19	1.00
	Permethrin	25.7 (18.9–35.9)	198.7 (117.4–454.9)	1.5 (± 0.9)	14.0	19	0.78

^†^Confidence Interval.

^‡^Degrees of freedom.

The mixtures of the major constituents were prepared for compound elimination assay, which notable stage-specific difference was observed between adults and larvae (Table 2). The mixture of the four major constituents exhibited statistically the same mortality to the original oil in both stages (*P* < 0.05), indicating those compounds were responsible for the overall activity of the oil. In adults, artificial mixtures lacking thymol showed reduced toxicity. Although eliminating individual *p*-cyemene from the artificial blends did not affect the overall toxicity, the three treatments which lack both thymol and *p*-cymene (α-pinene + linalool, α-pinene, and linalool) exhibited statistically further decrese of mortality, indicating the boosting effect of *p*-cymene on the toxicity of thymol against the adult houseflies (*F*_13,56_ = 92.77, *P* < 0.001). In contrast, the elimination of thymol resulted in the complete loss of toxicity (*F*_13,56_ = 128.92, *P* < 0.001) in larvae regardless of whether *p*-cymene was eliminated or not.

**Table 2:** Insecticidal activity of artificial mixtures of thyme oil in the compound elimination assay against larval and adult houseflies.

	Blended compounds^†^				Mortality (% ± SE)^‡^	
	Thymol	*p*-cymene	linalool	α-pinene	Adult	Larvae
Artificial mixture	+	+	+	+	92 ± 3.7A	92 ± 5.8a
		+	+	+	44 ± 5.1B	4 ± 2.4b
	+		+	+	90 ± 3.2A	88 ± 5.8a
	+	+		+	92 ± 3.7A	92 ± 4.9a
	+	+	+		94 ± 4.0a	88 ± 4.9a
			+	+	2 ± 2.0c	0 ± 0.0b
		+		+	34 ± 7.5b	0 ± 0.0b
		+	+		38 ± 4.9b	0 ± 0.0b
	+			+	90 ± 4.5a	90 ± 3.2a
	+		+		94 ± 2.4a	92 ± 5.8a
	+	+			94 ± 4.0a	90 ± 5.5a
				+	2 ± 2.0c	0 ± 0.0b
			+		4 ± 2.4c	2 ± 2.0b
	+				90 ± 3.2a	86 ± 5.1a
Thyme oil					88 ± 2.2a	92 ± 1.2a

^†^Artificial oils were prepared by blending the compounds as marked ( +) and applied at the equivalent dose of LD_90_ of the original thyme oil.

^‡^Com Different letters indicate statistical differences based on ANOVA and Tukey post hoc test (*P* < 0.05).

Dose–response assays were used to further characterize the synergy between thymol and *p*-cymene (Table 3). In adults, the topical toxicity of thymol was 2.2 times higher than that of *p*-cymene (LD_50_ = 121.4 μg/insect vs. 270.4 μg/insect). When blended in their natural proportion (4.6:5.4), the two compounds interacted synergistically with an R value of 1.7 (observed LD_50_ = 101.1 μg/insect and expected LD_50_ = 172.8 μg/insect). Likewise, they were synergistic in other ratios (*R* = 1.6, 2.0, and 1.98 for [thymol:*p-*cymene] 1:1, 1:2, and 1:4, respectively). Otherwise, they were merely additive at ratios of 1:9, 2:1, 4:1, and 9:1. In larvae, thymol was 10.7 times more toxic than *p*-cymene (LD_50_ = 80.6 μg/insect and 864.3 μg/insect, respectively) and blending them showed either antagonistic (1:2, 4.6:5.4, 1:1, 2:1, and 4:1) or additive effects (1:9, 1:4, and 9:1).

**Table 3:** Insecticidal activity of thymol and *p*-cymene mixtures via topical application of different blending ratios in adult and larval houseflies.

Stage	Proportion		Observed LD_50_ (μg/insect, 95% CI^†^)	Slope (± SE)	*χ*^*2*^	*d.f*	*p*	Expected LD_50_ (μg/insect, 95% CI^†^)	*R*	Note
	Thymol	*p*-Cymene								
Adult	1.00	0	121.4 (105.9–140.0)	3.4 (± 0.4)	25.4	22	0.28			
	0	1.00	270.4 (249.5–296.3)	5.2 (± 0.6)	11.8	22	0.96			
	0.10	0.90	223.0 (182.9–274.1)	2.5 (± 0.3)	7.7	16	0.96	240.8	1.08	Add
	0.20	0.80	109.7 (99.5–121.3)	4.6 (± 0.5)	12.2	22	0.95	217.1	1.98	Syn
	0.33	0.67	97.84 (83.5–115.5)	2.7 (± 0.3)	19.5	22	0.62	192.1	1.96	Syn
	0.46	0.54	101.1 (92.6–110.3)	5.5 (± 0.6)	16.3	19	0.64	172.8	1.71	Syn
	0.50	0.50	101.9 (92.9–112.5)	5.0 (± 0.6)	19.2	19	0.45	167.6	1.65	Syn
	0.67	0.33	146.1 (123.9–173.8)	2.5 (± 0.3)	18.8	25	0.81	148.9	1.02	Add
	0.80	0.20	158.2 (144.1–175.2)	5.2 (± 0.7)	10.4	16	0.84	136.4	0.86	Add
	0.90	0.10	107.8 (92.0–126.8)	2.9 (± 0.4)	13.4	19	0.82	128.5	1.19	Add
Larva	1.00	0	80.6 (67.8–95.9)	2.5 (± 0.3)	20.8	22	0.54			
	0	1.00	864.3 (807.2–925.6)	6.6 (± 0.7)	17.8	22	0.72			
	0.10	0.90	590.4 (543.5–642.7)	6.2 (± 0.8)	12.2	16	0.7	438.3	0.74	Add
	0.20	0.80	486.9 (419.9–570.2)	3.1 (± 0.4)	10.6	19	0.94	293.6	0.60	Add
	0.33	0.67	609.1 (545.9–682.4)	3.6 (± 0.4)	18.8	25	0.81	204.1	0.34	Ant
	0.46	0.54	499.9 (406.4–634.1)	2.3 (± 0.3)	12.9	19	0.84	158.0	0.32	Ant
	0.50	0.50	445.3 (355.9–574.8)	2.0 (± 0.3)	14.8	19	0.73	147.5	0.33	Ant
	0.67	0.33	358.2 (313.5–410.4)	3.0 (± 0.3)	17.5	26	0.89	115.7	0.32	Ant
	0.80	0.20	223.9 (192.6–263.0)	3.2 (± 0.5)	12.5	16	0.71	98.5	0.44	Ant
	0.90	0.10	113.3 (92.1–141.7)	2.2 (± 0.3)	10.6	19	0.94	88.7	0.78	Add

^†^Confidence Interval.

### Divided application and injection assay

To probe whether the observed synergy occurred due to increased cuticular penetration, divided application and injection assays were performed (Table 4). No synergy was observed when the two compounds were administered separately on different parts of the thorax. In injection assays, the injected compounds were more toxic than when topically applied, as they bypassed the cuticular barrier. For thymol, toxicity increased 7.9 times when injected (LD_50_ = 15.3 μg/insect vs. 121.4 μg/insect), although it was only 1.7 times higher for *p*-cymene (LD_50_ = 159 μg/insect vs. 270.4 μg/insect). However, when the mixture was injected, no synergy was observed. Rather, the combination was antagonistic when blended in a 1:2 ratio (*R* = 0.5).

**Table 4:** Bioassay method-dependent insecticidal activity of selected synergistic thymol and *p*-cymene mixture in adult houseflies.

Method^†^	Proportion		Observed LD_50_ (μg/insect, 95% CI^‡^)	Slope (± SE)	*χ*^*2*^	*d.f*	*p*	Expected LD_50_ (μg/insect, 95% CI^‡§^)	*R*	Note
	Thymol	*p*-Cymene								
DIV-A	0.46	0.54	236.5 (203.2–275.5)	2.9 (± 0.3)	12.4	22	0.95	172.8	0.73	Add
	0.33	0.67	214.9 (180.7–254.4)	2.5 (± 0.3)	10.0	22	0.99	172.8	0.80	Add
DIV-B	0.46	0.54	219.5 (197.5–242.7)	4.1 (± 0.4)	11.5	28	1.00	192.4	0.88	Add
	0.33	0.67	230.7 (207.6–255.7)	3.9 (± 0.4)	17.8	28	0.93	192.4	0.83	Add
INJ	1.0	0	15.3 (13.2–17.8)	3.0 (± 0.3)	12.5	22	0.95			
	0	1.0	159.0 (136.2–186.4)	2.8 (± 0.3)	13.9	22	0.90			
	0.46	0.54	55.8 (50.5–61.6)	4.5 (± 0.5)	7.4	22	1.00	29.9	0.54	Add
	0.33	0.67	78.2 (68.5–89.0)	4.0 (± 0.5)	6.9	16	0.98	38.8	0.50	Ant

^†^DIV-A: thymol and *p*-cymene were applied separately on the thoracic notum and sternum, respectively, or DIV-B: on the sternum and thoracic notum, respectively. INJ: thymol and *p*-cymene were injected individually or as binary mixtures.

^‡^ Confidence Interval.

^§^Expected LD_50_ values in the separate applications were calculated based on the topical LD_50_ values presented in Table 3.

### Sequential, large-volume treatment, and contact angle measurement

Sequential treatments were conducted to evaluate whether administering thymol and *p*-cymene at hourly intervals affects their penetration route. Table 5 shows that there was no significant interaction between the compounds when thymol was administered before *p*-cymene ((*R* = 0.6 at 4.6:5.4; *R* = 0.5 at 1:2) or vice versa (*R* = 0.6 at 4.6:5.4; *R* = 0.7 at 1:2). Additionally, no statistical difference in the contact angles of the wax layer by thymol or *p*-cymene was observed compared to blank and acetone application (*P* > 0.05), indicating that the synergistic effect between the two compounds may not be due to a disturbance on the integrity of cuticular wax layer (See Supplementary Fig. S3 for further details).

**Table 5:** Sequential treatment assay for detecting the potential effects of thymol or *p*-cymene to facilitate internal penetration route for the other as well as for a large volume treatment in adult houseflies.

Treatment scheme^†^	Proportion		Observed LD_50_ (μg/insect, 95% CI^‡^)	Slope (± SE)	*χ*^*2*^	*d.f*	*p*	Expected LD_50_ (μg/insect, 95% CI^‡§^)	*R*	Note
	Thymol	*p*-Cymene								
TC	0.46	0.54	292.7 (251.9–340.0)	3.3 (± 0.5)	10.0	16	0.87	172.8	0.59	Add
TC	0.33	0.67	356.4 (322.8–394.1)	4.1 (± 0.4)	16.7	28	0.95	192.1	0.54	Add
CT	0.46	0.54	304.4 (276.6–336.7)	4.5 (± 0.5)	13.2	22	0.93	172.8	0.57	Add
CT	0.33	0.67	273.1 (237.9–313.7)	3.5 (± 0.4)	15.2	19	0.71	192.1	0.70	Add
LV	1.0	0	89.8 (77.6–103.7)	3.1 (± 0.3)	5.7	22	1.00			
LV	0	1.0	223.4 (194.2–258.9)	3.5 (± 0.5)	12.2	16	0.73			
LV	0.46	0.54	182.9 (144.1–233.6)	1.8 (± 0.2)	12.2	22	0.95	132.6	0.72	Add
LV	0.33	0.67	239.6 (202.2–281.2)	2.8 (± 0.4)	9.3	19	0.97	149.8	0.63	Add

^†^TC: thymol was applied first, followed by *p*-cymene application, CT: compounds were applied in the order of *p*-cymene and thymol, LV: large-volume treatment (1.5 μL).

^‡^Confidence Interval.

^§^Expected LD_50_ values in the separate applications were calculated based on the topical LD_50_ values presented in Table 3.

The large-volume treatment indicated that administering 1.5 μL of thymol and *p*-cymene mixture instead of 0.5 μL completely wet the surface and did not result in a synergistic effect (*R* = 0.7 at 4.6:5.4; *R* = 0.6 at 1:2), suggesting that the increased penetration of the compounds may be due to the greater wetting of the surface when the two compounds were mixed. Therefore, the results indicate that the increase in the wetting area can be responsible for the observed synergy.

### GC–MS analyses of in vivo penetration

The penetration-enhancing effect of *p*-cymene on thymol was confirmed by comparing the in vivo penetration amounts at 1 h post-treatment in adults and larvae with thymol and *p-*cymene individually and with a mixture thereof. In adults, the internal amount of thymol significantly increased when administered within the mixture compared to that when administered alone (Fig. 2a, *df* = 4, *P* = 0.005), but that was not the case for *p*-cymene. In the larvae (where no synergy was observed), the penetration of both compounds did not differ between administration treatments (Fig. 2b). Furthermore, when the adults were treated with larger volumes of the test solutions (1.5 μL), the penetration amount of both compounds did not differ between administration treatments, as predicted in the bioassay (Fig. 2c). Notably, despite the application of the same dose of thymol and *p*-cymene, penetration significantly increased when a larger volume was used during the individual (Fig. 2a and c; *df* = 4, *P* = 0.022 for thymol; *df* = 4, *P* = 0.042 for *p*-cymene) and combined treatments.

### Toxicity and penetration to flies with modified epidermis

**Figure 1:**
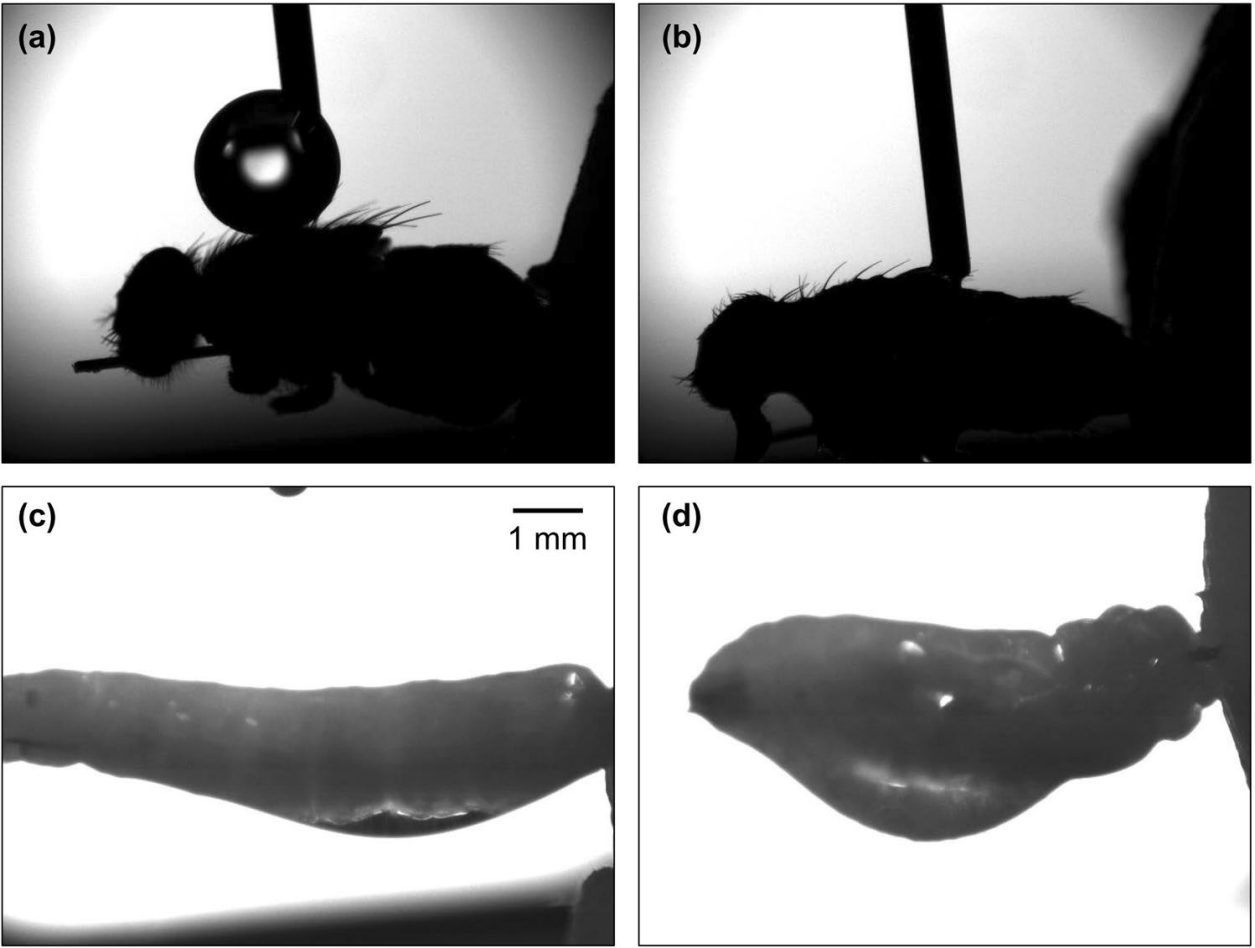
Change of surface wetting properties in dewaxed adults and artificial wax-coated larvae. Four microliters of deionized water were applied using a pendant-drop method. Intact adults showed superhydrophobicity (**a**) but became wettable after rinsing with hexane (**b**). Intact larvae showed moderate hydrophilicity (**c**) while being more hydrophobic after being coated with artificial wax (**d**).

To evaluate the synergistic role of the cuticular wax layer, the epidermal wetting properties were artificially interchanged (Fig. 1 and Table 6). The adults devoid of the hydrophobic wax layer (DA) were 2.1 and 1.4 times more susceptible than intact adults (IA) to thymol and *p*-cymene, respectively (LD_50_, DA: 57.8 μg/insect, IA:121.4 μg/insect for thymol; DA:189.4 μg/insect, IA: 270.4 μg/insect for *p*-cymene). In contrast, the larvae artificially coated with long-chain hydrocarbon (n-eicosane) (WL) were 3.2 and > 2.0 times more tolerant than intact larvae (IL) to thymol and *p*-cymene, respectively (LD_50_, WL: 261.3 μg/insect, IL:80.6 μg/insect for thymol; WL: > 1714.0 μg/insect, IL:864.3 μg/insect for *p*-cymene). Both of the synergistic combinations in topical application on intact adult houseflies (thymol:*p*-cymene = 0.46:0.54, 0.33:0.67) failed to display synergistic toxicity on dewaxed adults with the R values of 0.66 and 0.77, respectively. On the other hand, whereas the two combinations were not synergistic against the intact larvae, the two mixtures were synergistic on the artificially wax-coated larvae, with the R values of 2.00 and 2.11, respectively. These changes in the interactions following wax layer modification indicate the crucial role of the wax layer in synergism.

**Table 6:** Synergistic effects of topical administration of thymol, *p*-cymene*,* and mixtures thereof on epidermally modified adult and larval houseflies.

Insect^†^	Proportion		Observed LD_50_ (μg/insect, 95% CI^‡^)	Slope (± SE)	*χ*^*2*^	*d.f*	*p*	Expected LD_50_ (μg/insect, 95% CI^‡^)	*R*	Note
	Thymol	*p*-Cymene								
DA	1.0	0	57.8 (49.5–67.7)	2.8 (± 0.3)	20.2	22	0.57			
	0	1.0	189.4 (169.0–212.8)	3.7 (± 0.5)	12.8	22	0.94			
	0.46	0.54	139.6 (114.2–173.5)	2.5 (± 0.3)	7.4	16	0.96	92.6	0.66	Add
	0.33	0.67	140.4 (123.5–159.9)	3.8 (± 0.4)	14.3	22	0.89	108.2	0.77	Add
WL	1.0	0	261.3 (210.6–327.8)	2.1 (± 0.3)	15.8	19	0.67			
	0	1.0	> 1714.0	0.0 (± 0.0)						
	0.46	0.54	241.3 (205.4–283.3)	2.9 (± 0.4)	19.8	19	0.41	481.8	2.00	Syn
	0.33	0.67	286.6 (236.7–351.0)	3.2 (± 0.3)	35.6	22	0.03	604.7	2.11	Syn

^†^DA: dewaxed adult flies, WL: artificial wax-coated larvae.

^‡^Confidence Interval.

**Figure 2:**
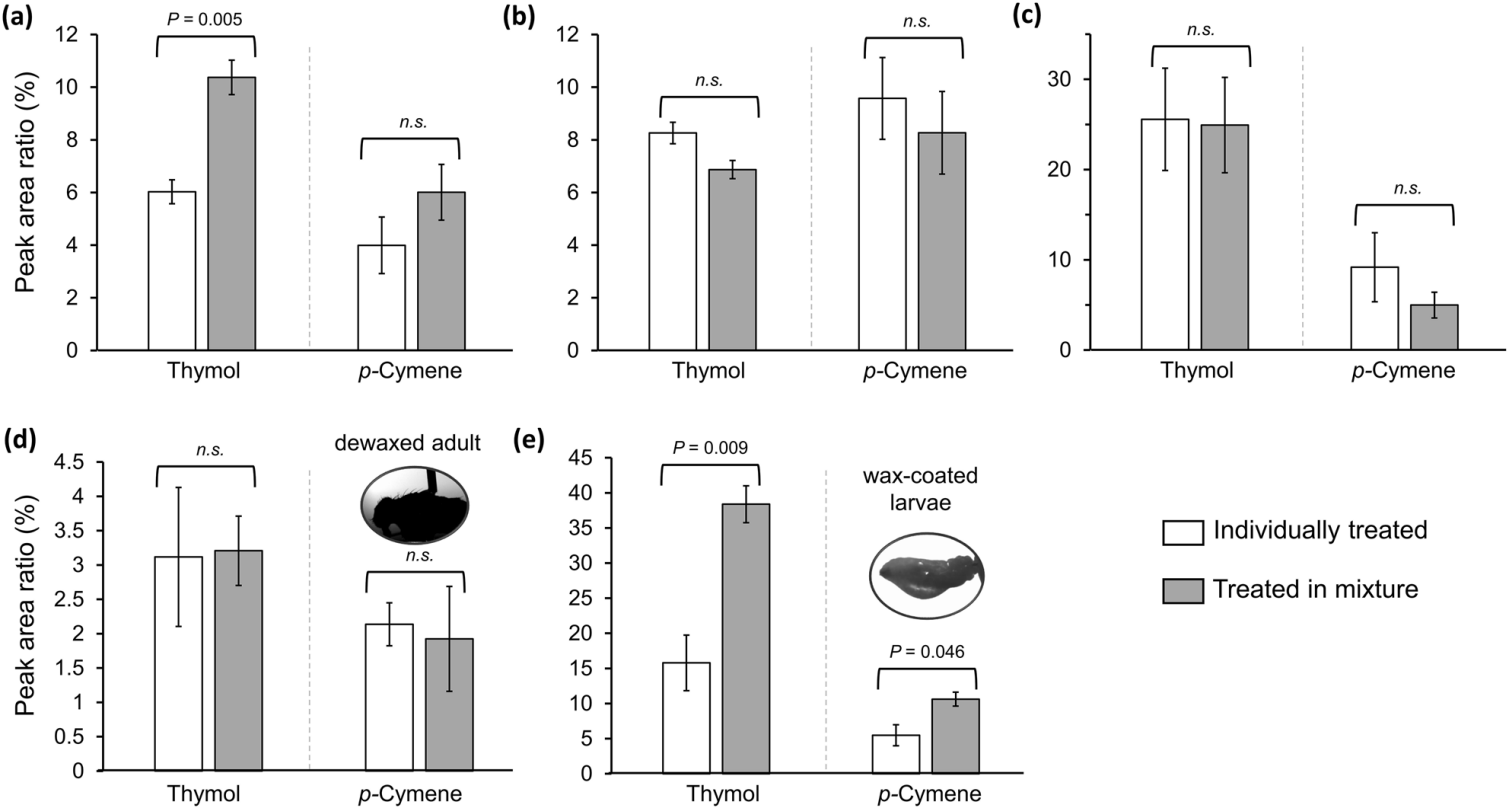
GC–MS analysis of cuticular penetration of thymol, *p*-cymene, and the mixture thereof after treating standard (0.5 µL) volume of test solutions onto adults (**a**) and larvae (**b**). The penetration in large-volume treatment (1.5 µL) was also investigated (**c**). When cuticualr properties were interchanged, the overall interaction was reversed correspondingly, as penetration amounts of both compounds were did not change in dewaxed adults (**d**), while increased in wax-coated larvae (**e**).

In accordance with these results, *p*-cymene did not enhance the in vivo penetration of thymol into the DA (Fig. 2d). Additionally, in the WL, the compounds penetrated more easily when administered as a mixture than when administered individually (Fig. 2e, *P* = 0.009 for thymol; *P* = 0.046 for *p*-cymene).

## Discussion

In the present study, we investigated the synergistic mechanism of the two major constituents of thyme oil, thymol and *p*-cymene, against the adult and larval stages of houseflies. Synergistic toxicity was observed only in adults, where *p*-cymene enhanced the cuticular penetration of thymol. Further bioassays revealed that the increased penetration was due to the expanded wetting area and not disruption of the protective layer. A comparison between wax-covered adults and wax-devoid larvae confirmed the crucial role of the epicuticular wax layer in this synergy.

Thyme oil has notable insecticidal properties and is one of the most frequently reported essential oils^26–29^. The chemical composition of essential oils can vary dynamically depending on the extraction method, genetic background (cultivar) of the source plant, and the abiotic (temperature and humidity) and biotic (density, herbivory, infection) stresses that the source plant is exposed to^30–34^. The chemical composition of the thyme oil used in this study differed from that of other studies^3,35^. Differences in the major constituents and their relative proportions can result in differences in insecticidal activity^36^. Moreover, it is well-known that not only the major constituents but also the minor ones can significantly contribute to the overall activity in the essential oils^37^.

Reports of enhanced toxicity in essential oil blends have been frequent in the literature^19,26,38,39^. However, compared to the plethora of reports on synergistic effects, presumably due to the diversity in chemical structures and compositions, a generalized understanding of their synergistic mechanisms is yet to be explored. For example, our study on adult houseflies showed that a binary mixture of thymol and *p*-cymene was synergistic in natural (4.6:5.4, w/w), 1:1, 1:2, and 1:4 ratios but not at 9:1, 4:1, 2:1, or 1:9, indicating composition-dependent interactions in synergy. Two categories of synergistic interactions have been proposed: internal and external. Previous studies have shown that synergistic compounds can modulate nerve sensitivity to boost the toxicity of the primary active compounds. This is classified as an internal interaction^18,40,41^. A recent study found that internal synergy may not be restricted to neurotoxic insecticides but also to respiratory blockers^42^. In contrast, the synergistic combination in the present study was not synergistic when injected into the adults, suggesting that it is an external effect. Moreover, the divided topical application failed to increase toxicity, indicating that synergy between thymol and *p*-cymene only occurs when they are co-applied as a mixture. Several previous studies have proposed that enhanced cuticular permeation could be an external synergistic mechanism of a toxicokinetic^3,19,43,44^. We demonstrated that thymol recovery from the adult lymphal extracts increased in response to administration of the thymol/*p*-cymene mixture but not from the larval lymphal extract. This strong correlation with the bioassay results confirmed *p*-cymene’s role as a penetration enhancer in synergistic interactions.

It was previously hypothesized that *p*-cymene disrupts the physicochemical integrity of the cuticular layer in the cabbage looper *Trichoplusia ni*^3^. Generally, cuticular wax layers are considered a barrier to insecticide penetration. For instance, insecticide toxicity increases when the wax layer of adult houseflies is abraded using chloroform, possibly because of increased penetration^45^. A recent study also showed that rosehip oil containing 1,8-cineole and camphor dissolves the protective wax bloom of the scale insect *Icerya aegyptiaca, *thereby boosting the penetration of toxic matrine^44^. As shown in this study, wax layer seems to work as a barrier for toxicants that increased toxicity was observed when the wax layer was removed in adults and decreased mortality was found on artificially wax-coated larvae. Disruption of the wax layer by *p*-cymene might be suggested as the potential mechanism of synergy on the toxicity of thymol in the adult houseflies. However, our study suggests that *p*-cymene may not affect the integrity of wax layer, as no increased toxicity of thymol was found in the sequential application. Rather, better spreading of thymol by the addition of *p*-cymene due to its low hydrophobicity of *p*-cymene would be more compelling. Administering thymol in a larger volume of solvent increased its toxicity (from 121.4 to 89.8 μg/insect of LD_50_), and the combination in a larger volume showed neither synergistic toxicity nor increased cuticular penetration. These observations indicated that *p*-cymene spreads through thymol to cover a larger area than it can by itself, stimulating the simultaneous penetration of more molecules through the waxy cuticle.

Owing to their small size, insects are highly vulnerable to water loss, and their cuticular wax layers play a crucial role in protecting them from desiccation. Tolerance to desiccation varies with water availability because insects from arid habitats have less cuticular transpiration than mesic insects^46–48^. The general waterproofing mechanism of cuticular wax is quantitatively and qualitatively diverse^49^. Notably, it has been demonstrated that the number of saturated hydrocarbons in the wax layer is negatively correlated with water loss^50^. An increase in n-alkanes in the wax layer during the warm season has been observed in several species, including *Centruroides sculpturatus*, *Eloedes armata,* and *Cicindela obsoleta*^46,51,52^. Although houseflies have a shorter lifespan and do not exhibit seasonal changes, differences in cuticular wax composition have been observed between adults and larvae. The adults are terrestrial and have a greater need to prevent water loss, while larvae live in a mesic habitat where water loss is less significant^25,53^. Although no analysis of cuticular wax composition was conducted in the present study, the difference in hydrophobicity was indisputable when observing the behavior of water droplets on the cuticle (Fig. 1a and c). When the cuticular hydrophobicity of adults and larvae was manipulated, the bioassay results changed accordingly, indicating that cuticular hydrophobicity plays a role in synergy. These results highlight the stage-specific differences in susceptibility to insecticidal compounds and suggest that further research on the toxicokinetics of insecticides in terms of cuticular properties could benefit pest management strategies.

Because the prolonged use of conventional insecticides has led insect pests to develop resistance traits, studies have suggested that cuticular thickening by overproduction of the wax layer can confer resistance by slowing insecticide penetration^54,55^. In resistant strains bearing such mechanisms, the search for efficient penetrating synergists can help overcome the problem of resistance. However, further experimental evidence is required to support this hypothesis.

To conclude, we hypothesized enhanced cuticular penetration as the potential synergistic mechanism of the two major constituents of thyme oil, thymol and *p*-cymene, in housefly, and the difference in cuticular composition as well as hydrophobicity of the surface would determine the degree of penetration and toxicity. Our comprehensive study clearly shows the stage-dependent difference in the synergistic toxicity along with the cuticular permeation of the compounds. By examining the surface chemistry of target pests, more effective pest control strategies using penetration-enhancing synergists can be developed.

## Materials and methods

### Test insects

The susceptible strain of houseflies was obtained from the Korean Disease Control and Prevention Agency (KCDC) and was maintained in the insectary of Seoul National University. The colony had not been exposed to any known pesticides for over nine years. Hatched larvae were fed a mixture of rodent diet (Purina 38,057; Purina, St. Louis, MO, USA), oak sawdust, and distilled water at a weight ratio of 9:1:10. Pupae were transferred to meshed cages (30 × 30 × 30 cm), and the emerged adults were provided with a 10% sucrose solution. Insects were reared under conditions of 26 ± 2 °C, 20 ± 5% RH, and a 12:12 (L:D) photoperiod.

### Test oil and chemicals

Steam-distilled thyme essential oil extracted from the flowering tops and leaves of *Thymus vulgaris* (L.) was purchased from Dr. Mercola® (Cape Coral, FL, USA). The plant was grown via USDA-organic certified method, according to National Organic Program (NOP), and the use of plant parts in the study complies with international, national, and/or institutional guidelines. Artificial wax (n-eicosane, 99%) and four of the major constituents of thyme oil, namely thymol (> 98.5%), *p*-cymene (99%), linalool (97%), and α-pinene (98%), were obtained from Sigma-Aldrich (St. Louis, MO, USA). Acetone (99.5%) and n-hexane (95.0%) were purchased from Daejung Chemicals (Siheung-si, Gyeonggi-do, South Korea). Technical grade permethrin (95.9%) was provided by LG Chem (Daejeon, South Korea) and used as a positive control.

### Topical application

Late third-instar larvae were used to examine the larvicidal activity of the selected oils and compounds. Ten larvae were fastened to a laboratory-designed apparatus (Supplementary Fig. S1). Each larva (in a holding tube) received 3 μL of test solution on the thoracic segments and was then dried for 1 h. Larvae were gently pulled from the tethers and transferred to a 385 mL cup with 10 g of larval feed for observation.

Adulticidal contact toxicity was evaluated in female flies at 5–10 d post-emergence. A group of ten flies was anesthetized using medical-grade CO_2_ and placed on a chill plate. The test oil and compounds were diluted in acetone, and 0.5 μL of the test solution was topically applied onto the thoracic notum of the fly using a Hamilton Microliter syringe (Hamilton Company, Reno, NV, USA) fitted with a repeating dispenser. After the solvent was evaporated, the treated insects were then transferred to clean cups for observation.

The treated flies were maintained in the observation conditions for 24 h, after which mortality was recorded and LD_50_ values were determined. The tests were repeated three to four times.

### Compound elimination assay and synergy determination

GC–MS analysis indicated that *p*-cymene (38.0%), thymol (31.8%), linalool (4.1%), and α-pinene (3.1%) were the major constituents of thyme oil (Supplementary Table S1 and Fig. S2). A compound elimination assay was performed to evaluate the contribution of each compound to the overall insecticidal activity of the oil^37^. Briefly, a series of artificial oils were prepared using the four major constituents in their natural proportions, either as a full mixture or by omitting the target compounds. This resulted in 10 artificial oils. Each artificial oil was topically applied at the dose equivalent to the LD_90_ of thyme oil to both larvae and adults, as mentioned above, and the major active constituents were identified: thymol and *p*-cymene. All further experimental procedures focused on these two active ingredients.

In the following experiment, binary mixtures of 10 different ratios were prepared, and their ratio-dependent interactions were further investigated. The LD_50_ values of the mixtures were estimated, and their interactions were determined based on Wadley’s model^56^. The expected LD_50_ was calculated as follows:

$${Expected LD}_{50}=\frac{a+b+c+ \cdots n}{\frac{a}{{LD}_{50}A} + \frac{b}{{LD}_{50}B} + \frac{c}{{LD}_{50}C} + \cdots + \frac{n}{{LD}_{50}N}}$$

where *a* is the proportion of compound A in the mixture and *LD*_*50*_*A* represents the observed LD_50_ of compound A, and so forth. The synergy ratio (R) was calculated as follows:

$$R= \frac{expected {LD}_{50} of the mixture}{observed {LD}_{50} of the mixture}$$

where the interaction was defined as either synergistic (R > 1.5), additive (1.5 ≥ R > 0.5), or antagonistic (R ≤ 0.5).

### Divided application, injection, sequential application, and large-volume treatment

Previous studies have suggested two bioassay methods to distinguish the penetration-enhancing effect (as a synergistic mechanism) from other physiological interactions in a lepidopteran insect: a divided application assay and an injection assay^19,43^. The divided application method was performed by simultaneously applying thymol to the sternum and *p*-cymene to the thoracic notum (or vice versa) of the female flies. For the injection assay, 1 μL of test solution (either individual compounds or the binary mixture) was injected into the dorsal thorax of the adults using a manual microsyringe pump (DMP, World Precision Instruments, Sarasota, FL, USA), and the LD_50_ values after 24 h were determined. Two binary mixtures were used for this and all subsequent procedures, namely thymol:*p*-cymene 0.46:0.54, which reflects the relative proportions of the compounds in the tested thyme oil, and 0.33:0.67 (or 1:2), which reflects the ratio that showed highest synergy (*R* = 1.96) in topical application.

Two additional topical application assays were conducted to further investigate the penetration-enhancing effects. The first assay examined whether a preceding compound could facilitate the permeation of another by applying both compounds individually to the same spot within 1 h of each other. In the second assay, the volume of the application was increased from 0.5 to 1.5 μL, thereby treating the entire body of the adult fly.

### Contact angle measurement

To examine the impact of test compounds and solvent on the cuticular wax layer, contact angles of the treated surface were measured. Thymol and *p*-cymene were topically applied at their LD_50_ doses on the thorax of the intact adult houseflies as mentioned above, using acetone as a negative control. After an hour of incubation, the houseflies were anesthetized with CO_2_. One to three microliters of deionized water were applied on the treated surface and the contact angles were measured using SmartDrop Plus. This experiment was repeated five times.

### Cuticle modification assay

To address the role of the cuticular wax layer in the penetration-enhancing effects, we intentionally manipulated the wax layer. In adults, the thoracic notum was gently brushed several times using cotton swab soaked in n-hexane to remove the hydrophobic wax layer. The larvae were individually dipped into a saturated n-eicosane solution for 2 s to cover them with an artificial wax layer. The dewaxed adults and artificial wax-coated larvae were subjected to the same topical application as the intact individuals. The change in hydrophobicity resulting from the manipulation processes was confirmed by dropping 5 μL of deionized water on the treated surface using SmartDrop Plus (Femtofab, Sungnam, Gyeonggi, South Korea; Fig. 1).

### GC–MS analyses of thyme essential oil and lymphal extracts

The major constituents of thyme oil and the cuticular penetration of thymol and *p*-cymene were analyzed using a Chromatec-Crystal 9000 mass spectrometer (Chromatec, Mari El, Russia) equipped with an Agilent J&W VF-5 ms column (60 m, 0.25 mm ID and 0.25 μm thickness; Agilent Technologies, Santa Clara, CA, USA) and a Chromatec-Crystal (Chromatec). The injection volume was 1 μL, and helium (99.999%) with a flow rate of 1.5 mL/min was used as the carrier. The initial oven temperature was set at 50 °C for 5 min, then gradually increased to 65, 120, 180, 210, and 305 ˚C at increasing rates of 10, 5, 5, 5, and 20 °C /min, respectively. The temperatures were maintained for 30, 10, 0, 10, and 5 min. The data were analyzed using the NIST MS Search 2.4 libraries. To confirm the chemical analysis of thyme oil, an artificial oil was prepared by blending five of the major constituents (> 2% in composition) in their natural proportion and reanalyzed under the same condition above.

The relative cuticular penetration of thymol, *p*-cymene, and their binary mixtures (1:2) was also examined. Insects were treated with the LD_50_ values of the individual compounds. The treated insects were incubated for an hour and rinsed with n-hexane by gentle swirling. Insects were then transferred into a 2 mL microcentrifuge tube filled with 1.5 mL of n-hexane and homogenized using a Bead Beater homogenizer (Minilys, Bertin Instruments, Montigny-le-bretonneux, Ile-de-France, France). The crude extracts were filtered and immediately analyzed using GC–MS. The analyses were repeated three times.

### Statistics

The LD_50_ values of the test compounds and oil were determined using probit analysis. Differences between groups in the compound elimination assay were examined using one-way ANOVAs with Tukey post hoc tests. Student’s t-tests were used to compare cuticular penetration between individually-treated and mixture-treated compounds. All statistical analyses were performed using SPSS version 25 (IBM Corp., Armonk, NY, USA).

### Supplementary Information

Supplementary Information.

## Data Availability

The data that support the findings of this study are available from both authors, J.Y and J.-H. T., upon reasonable request.
