# Supplementary material for: Cuticular property affects the insecticidal synergy of major constituents in thyme oil against houseflies, Musca domestica
Source: Sci Rep. 2023 Aug 4;13:12654. doi: 10.1038/s41598-023-39898-6 (PMC10403520; doi:10.1038/s41598-023-39898-6)
Supplement: Supplementary file 1 — Supplementary Information. [file 41598_2023_39898_MOESM1_ESM.pdf]

Supplementary Information

Cuticular property affects the insecticidal synergy of major constituents in thyme oil against houseflies, *Musca domestica*

Junho Yoon[1] and Jun-Hyung Tak[1,2*]

[1]Department of Agricultural Biotechnology, Seoul National University, Seoul 08826, South Korea

[2]Research Institute of Agriculture and Life Sciences, Seoul National University, Seoul 08826, South Korea

[*]Corresponding author (jhtak@snu.ac.kr)

Supplementary Figure S1. The laboratory apparatus designed to topically apply test solutions to housefly larvae. The end of a micropipette tip was cut to form a point. The larvae were inserted from the top and then gently secured by pulling them outwards through the cut-off tip. The holes were spaced 15-mm apart.

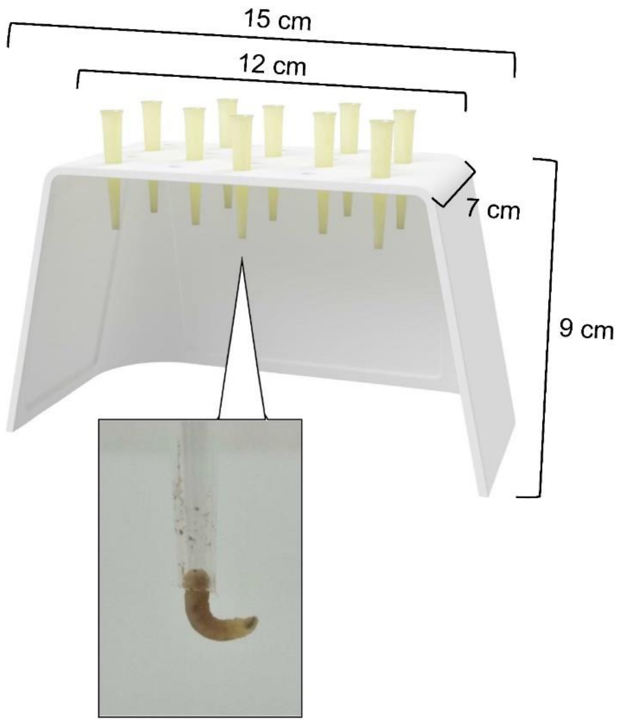

Supplementary Table S1. Chemical composition of thyme essential oil

Compounds	Retention time (min)	Retention Index	Composition (%)
α-Pinene	22.68	1026	3.10
Bicyclo-heptane	24.64	1039	1.07
Camphene	24.87	1041	2.80
3-Carene	35.09	1112	1.62
P-cymene	38.59	1140	38.02
Linalool	46.21	1200	4.13
α-Terpineol	52.70	1297	1.05
Thymol	60.36	1393	31.81
Total identified			83.6

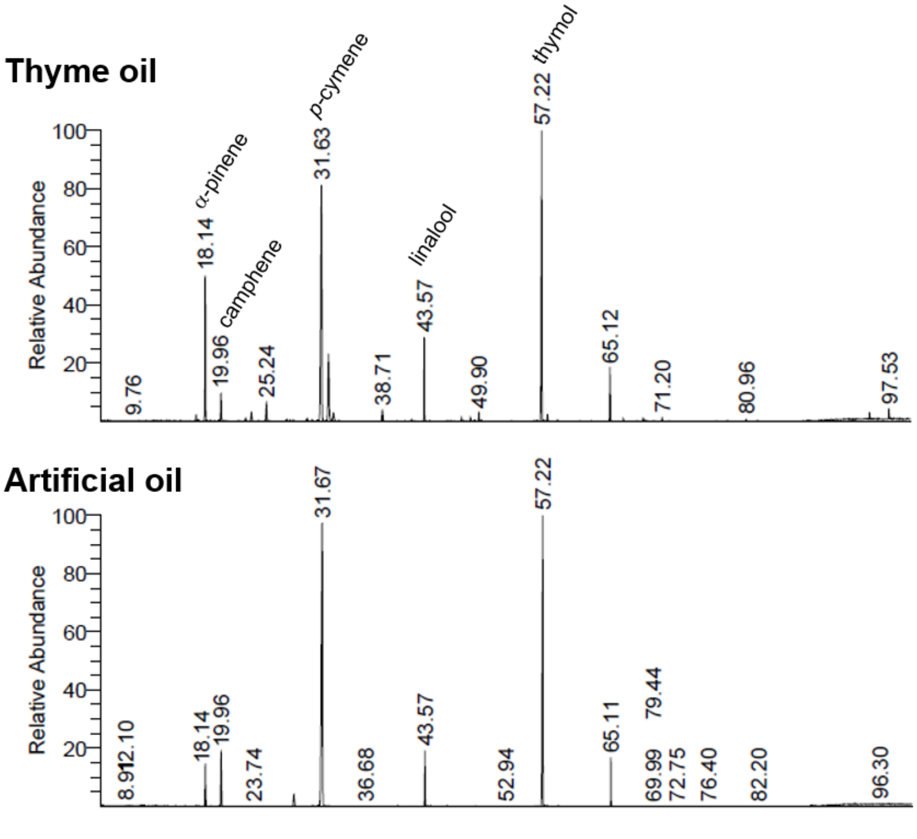

Supplementary Figure S2. GC-MS chromatograms of thyme oil and artificial oil containing five major constituents of thyme oil.

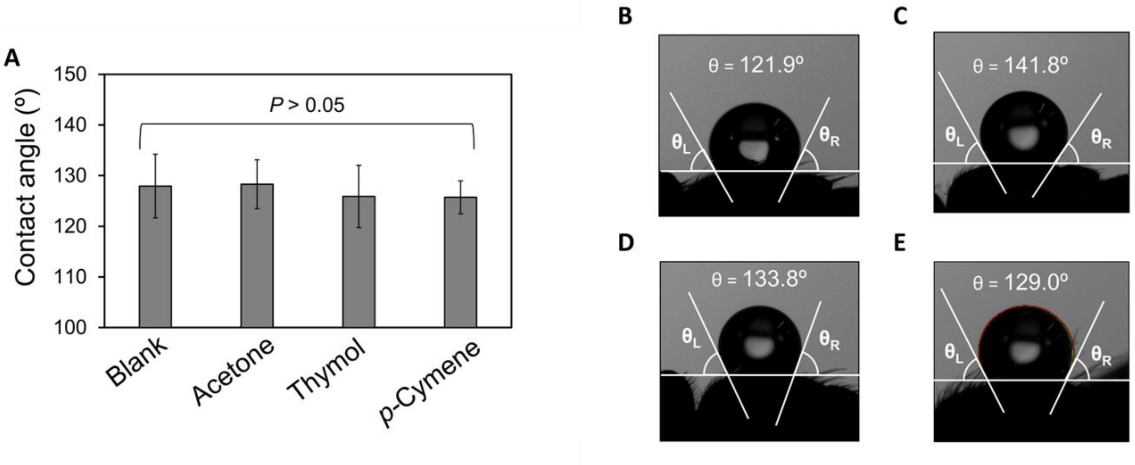

Supplementary Figure S3. Contact angles of test compounds-treated surface on the thorax of adult houseflies. Representative photographs of contact angles are shown for the following treatments: blank (no treatment, B), acetone (C), thymol (D), and p-cymene (E). The contact angle (A) is the mean value of the left angle (θ_L) and the right angle (θ_R).
